# Clinical Implications of Girdin Protein Expression in Glioma

**DOI:** 10.1155/2013/986073

**Published:** 2013-10-27

**Authors:** Liwei Zhao, Shuyin Ma, Qing Liu, Peng Liang

**Affiliations:** ^1^Department of Neurosurgery, The First Affiliated Hospital of Harbin Medical University, Harbin, Heilongjiang 150001, China; ^2^Department of Rehabilitation, The First Affiliated Hospital of Harbin Medical University, Harbin, Heilongjiang 150001, China; ^3^Department of Neurosurgery, The Third Affiliated Hospital of Harbin Medical University, Harbin, Heilongjiang 150040, China

## Abstract

*Objective*. To investigate the expression status of Girdin in glioma and the relationship between Girdin expression and the biological behavior of glioma. *Materials and methods*. The expression status of Girdin in glioma from 560 cases was evaluated by RT-PCR, Western Blot and immunohistochemistry. The relationship between Girdin expression and clinic-pathological parameters as well as prognosis was also studied. *Results*. The expression of Girdin in high grade glioma was significantly higher than low grade glioma. After universal analysis, the expression of Girdin protein is closely related to KPS score, extent of resection, Ki67 and WHO grade, but it was not related to sex and age. Finally, extent of resection, Ki67 and WHO grade were indentified to be related to the Girdin protein expression in logistic regression. Interestingly, we found that the expression of Girdin is significantly related to the distant metastasis of glioma. After COX regression analysis, KPS score, Extent of resection, Ki67, WHO grade as well as Girdin were observed to be independent prognostic factors. *Conclusions*. Girdin is differential expressed in the glioma patients and closely related to the biological behavior of Glioma. Finally, Girdin was found to be a strong predictor for the post-operative prognosis.

## 1. Introduction

Glioma is a type of tumor that starts in the brain or spine, and the most common site of gliomas is the brain [1]. Glioma make up ~30% of all brain and central nervous system tumors and 80% of all malignant brain tumors [2]. Gliomas are further categorized according to their grade, which is determined by pathologic evaluation of the tumor. Low-grade gliomas [WHO grade II] are well differentiated; these are benign and portend a better prognosis for the patient. High-grade [WHO grade III-IV] gliomas are undifferentiated or anaplastic; these are malignant and carry a worse prognosis [3]. 

High-grade Gliomas are highly vascular tumors and have a tendency to infiltrate [4]. They have extensive areas of necrosis and hypoxia. Often, tumor growth causes a breakdown of the blood-brain barrier in the vicinity of the tumor. As a rule, high-grade Glioma almost always grow back even after complete surgical excision and so are commonly called recurrent cancer of the brain. On the other hand, low-grade Gliomas grow slowly, often over many years, and can be waited and followed up closely without treatment unless they grow and cause symptoms [5].

The prognosis for patients with Glioma is generally poor, and it is especially so for older patients. Of 10,000 Americans diagnosed each year with malignant Gliomas, about half are alive one year after diagnose and 25% after two years [6]. So, how to diagnose and treat carcinoma early becomes of a great need. Glioma is a heterogeneous disease embracing several different phenotypes with consistently different biological characteristics [7]. Finding out a new potential marker for Gliomas and understanding the clinical significance of them and relationships between them would be valuable for current antitumor therapies and the development of novel ones [8].

Currently, studies addressing the function and specific mechanism of Girdin in the biological behavior of Glioma are rare. Moreover, the relationship between Girdin protein expression and clinicopathological features of Glioma is still not clear. In the present study, we investigate the expression status and clinical implications of Girdin protein in Glioma in order to lay a foundation for managing Glioma.

## 2. Materials and Methods

### 2.1. Clinical Specimens and Experimental Materials

The paraffin specimens of 560 brain glioma cases were collected from Liaoning Provincial Tumor Hospital and Harbin Medical University from January 2001 to January 2010. These cases were used for testing immunohistochemical protein levels and for the analysis of prognosis. The average age of enrolled patients was 45.21 ± 6.23 years (ranging from 35 to 83 years). The study protocol was approved by the Ethics Committee of Harbin Medical University.

### 2.2. RT-PCR

Total RNA was isolated using Trizole according to manufacture's instruction. cDNAs were synthesized using Revert Aid TM first cDNA synthesis kit (Fermentas). Quantitative real-time PCR was performed using SYBR Green PCR Master Mix on Applied Biosystems 7500 Fast Real-Time PCR System. Primers used in this work were designed using Invitrogen's Oligo Perfec Designer and evaluated with Netc Primer from Premier biosoft [9]. The reaction conditions are: 95°C and 5 min, followed by 40 cycles of 94°C 15 s, 55.5, or 55.2°C 20 s. Melting temperature curve analyses were performed after PCR.

### 2.3. Western Blot Procedures

Protein concentrations were determined using BCA assay (Santa Cruz Biotech). Equal amounts of total proteins were loaded and separated by SDS-PAGE electrophoresis and transferred onto PVDF membrane. Blots were blocked using 5% fat-free milk in PBS at 37°C for 2 h, followed by 2 PBST washes. Primary antibodies were then added: Girdin antibody (1 : 500; santa cruz biotechnology, inc) and GAPDH antibody (1 : 800; santa cruz biotechnology, inc). Blots were incubated with primary antibodies at 4°C overnight and then washed four times with PBST before adding secondary antibody. HRP-conjugated anti-rabbit or anti-mouse or anti-goat IgG antibody was used as a secondary antibody and incubated with the blots for 1 h at room temperature followed by four washes with PBST. Protein bands were visualized with chemiluminescence, and the target protein quantity was determined by normalizing the densities of corresponding bands to those of the loading control bands (GAPDH).

### 2.4. Immunohistochemistry Procedures

Tumor tissue microarray blocks were freshly cut into 4 *μ*m thick sections. Sections were fixed on slides and dried for 12–24 hours at 37°C. Sections were subsequently deparaffinized in xylene and rehydrated through gradually decreasing concentrations of ethanol to distilled water. After antigen retrieval, sections were incubated for 60 min with the primary antibody. Following washings with PBS, sections were incubated for 30 min in the secondary biotinylated antibody (multilink swine anti-goat/mouse/rabbit immunoglobulin; Dako). Following washings, Avidin Biotin Complex (1 : 1000 dilution; Vector Laboratories) was then applied to the sections for 30–60 min at room temperature. The immunoreactive products were visualized by catalysis of 3, 3-diaminobenzidine (DAB) by horseradish peroxidase in the presence of H_2_O_2_ following extensive washing. Sections were then counterstained in Gill's hematoxylin and dehydrated in ascending grades of methanol before clearing in xylene and mounting under a coverslip.

For negative controls, sections were treated with 0.01 mol/L PBS instead of primary antibodies; for positive controls, normal breast tissue section staining was positive. The positive cells of Girdin protein were defined as those with clearly brown granules located in the cytoplasm of cells. Two hundred cells from two selected representative fields of each section were counted by two independent observers for the determination of their immunostaining intensity. Staining intensity was initially recorded on a 4-point scale: 0: no staining; 1: light brown; 2: brown; 3: dark brown. The extent of staining also was initially assessed on a 3-point scale: 0: <9% positive cells; 1: 10%~50% positive cells; 2: >50% positive cells. According to the above assessment criterion, the immunostaining results were classified into 0~2: low/loss expression of Girdin protein, and 3~6: high expression of Girdin protein. 

### 2.5. Statistical Analysis

All data were analyzed with SPSS statistics software (Version 13.0, Chicago, IL, USA). The relationships between Girdin and other parameters were studied using the chi-square test, Fisher's extract test, or independent *t*-tests. Disease-specific survival was analyzed using the Kaplan-Meier method. The log-rank test was used to analyze differences in survival. Multivariate analysis was performed using the Cox proportional hazards model selected in forward stepwise. A *P* value of less than 0.05 was considered statistically significant.

## 3. Results

### 3.1. Girdin Expression in Human Gliomas Tissues at mRNA and Protein Levels

**Figure 1:**
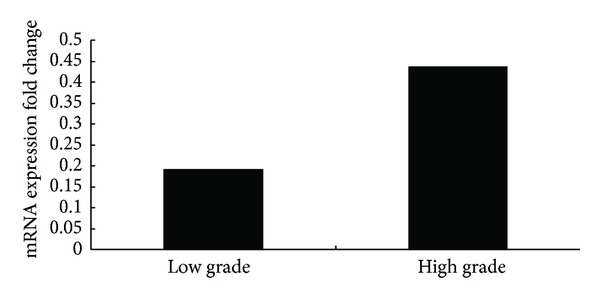
RT-PCR analysis showed that Girdin mRNA was upregulated in high-grade Gliomas cancer tissues when compared to low-grade tumor tissues (*P* = 0.001).

**Figure 2:**
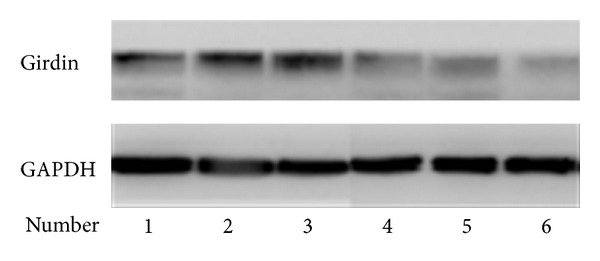
Girdin protein was upregulated in high-grade Gliomas cancer tissues when compared to low-grade tumor tissues in western blot analysis (*P* = 0.01).

RT-PCR analysis of Girdin mRNA expression in high-grade (WHO III-IV) and low-grade (WHO II) tumor tissues showed that Girdin mRNA was upregulated in high-grade Gliomas cancer tissues when compared to low-grade tumor tissues (*P* = 0.035) (Figure 1). Furthermore, in western blot analysis, the Girdin protein was upregulated in high grade gliomas cancer tissues when compared to low grade tumor tissues (*P* = 0.01) (Figure 2).

### 3.2. The Expression of Stem Cell Gene Girdin in Gliomas Patients and the Relationship between Girdin Expression and Clinic-Pathological Characteristics

**Figure 3:**
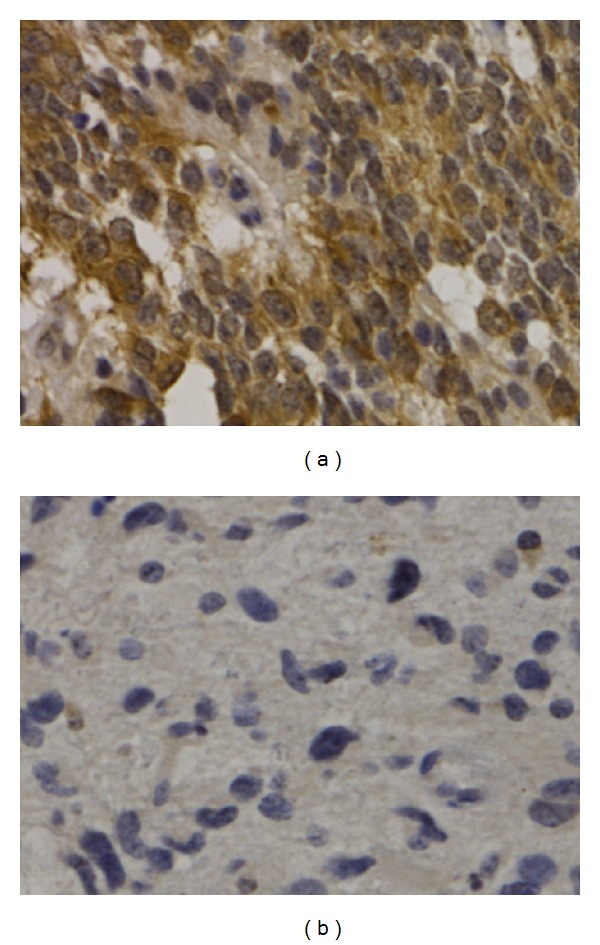
The expressions of Girdin in Gliomas tissues (×400). (a) High-grade gliomas stained with Girdin; (b) Low-grade gliomas stained with Girdin.

It was shown that Girdin was located in the cytoplasm of Glioma cells. In 174 (31.07%) Glioma patients, differently expressed Girdin protein in immunohistochemistry were observed (Figure 3). After universal analysis, it was found that the expression of Girdin protein is closely related to KPS score, extent of resection, Ki67, and WHO grade (*P* = 0.002, 0.001, 0.001, and 0.035, resp.), but it was not related to sex and age (*P* = 0.102 and 0.225, resp.) (Table 1). Finally, extent of resection, Ki67, and WHO grade were identified to be related to the Girdin protein expression in logistic regression (*P* = 0.011, 0.002, and 0.001) (Table 2).

### 3.3. Prognostic Analysis

**Figure 4:**
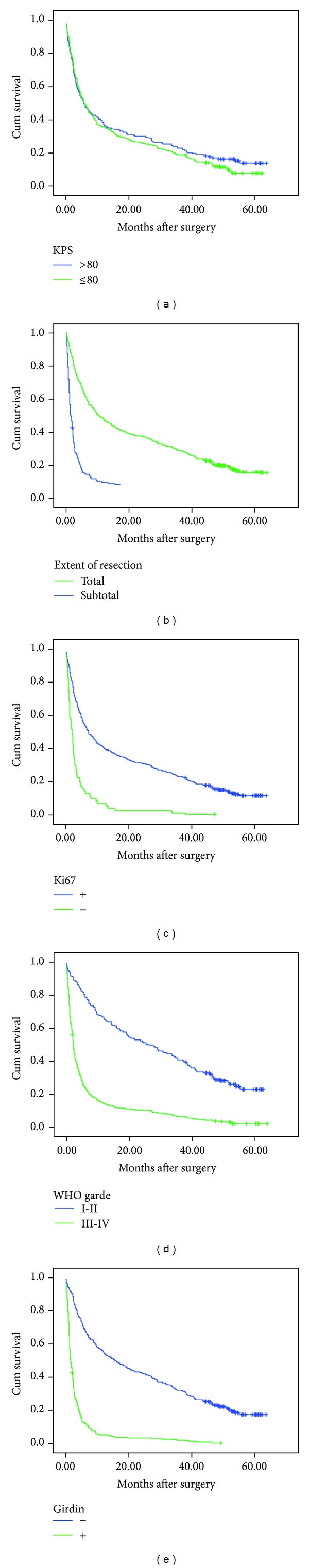
The survival analysis showed that KPS score (a), extent of resection (b), Ki67 (c), WHO grade (d), and Girdin (e) were observed to be independent prognostic factors (*P* = 0.044, 0.010, 0.002, 0.001, and 0.001, resp.).

 After survival analysis, the cases with highly expressed Girdin protein attained a significantly poorer postoperative disease-specific survival than those with high expressed Girdin protein (*P* = 0.001) (Figure 4). In the Cox regression test, KPS, extent of resection, Ki67, WHO grade, and Girdin were observed to be independent prognostic factors (*P* = 0.050, 0.001, 0.011, 0.001, and 0.001, resp.) (Table 3).

## 4. Discussion

Girdin is a novel protein, which is found at the crossroad of G protein signaling and tyrosine kinase receptor signaling [10]. It was also an actin-binding protein identified as a novel substrate of Akt, and it regulates the sprouting of axons and the migration of neural progenitor cells during early postnatal-stage neurogenesis in the hippocampus. When the epidermal growth factor receptor signaling is activated, Girdin is activated directly by Akt [11]. Recently, López-Sánchez et al. found that a Girdin-G*α*i molecular complex binds to the epidermal growth factor receptor and determines whether cells migrate or proliferate [12]. They also suggested that the expression of Girdin predicts patient survival in colon cancer and that Girdin may serve as a useful adjunct to traditional staging strategies in colorectal carcinoma [12]. Gliomas are the most common and aggressive type of brain tumor. Gliomas usually show hyperactivation of the PI3K-Akt pathway, a protumorigenic signaling cascade that contributes to pathogenesis [13]. Recently, Natsume et al. observed that stable Girdin knockdown in isolated Gliomas stem cells resulted in decreased expression of stem cell markers, including CD133, induced multilineage neural differentiation, and inhibited in vitro cell motility, ex vivo invasion, sphere-forming capacity, and in vivo tumor formation [14, 15]. They concluded that Girdin is required for Gliomas-initiating stem cells to sustain the stemness and invasive properties. However, there exists no studies investigating the expression status of Girdin protein in Gliomas, and its relationship to the biological behavior of Gliomas is still unclear. Furthermore, there is no study that addressed Girdin expression in Gliomas and the relationship between it and the prognosis of Gliomas.

The prognosis of Glioma was worse, though the patients received operations combined with radiotherapy and chemotherapy treatment [16]. Research priorities of the next 10 years will be committed to basic and translational medicine. The new treatment methods including the development of new drugs to block cell proliferation signaling pathway are to overcome the drug resistance of chemotherapy. In a study, Girdin is required for Glioma stem cells to sustain the stemness and invasive properties [17]. Therefore, Girdin maybe a potential new target for the treatment of Glioma.

In the study, we evaluated the expression status of Girdin in Glioma and observed that Girdin was significantly higher in Glioma tissues compared to paracancer tissues. After universal analysis, it was found that Girdin protein is closely related to KPS score, extent of resection, Ki67, and WHO grade. However, only extent of resection, Ki67, and WHO grade were identified to be related to the Girdin expression in multiple regression. Finally, Girdin was observed to be independent prognostic factor for glioma.

## 5. Conclusion

The expression of Girdin is closely related to the biological behavior of brain glioma. It is an independent prognostic factor in glioma, which can provide the basis for clinical treatment of brain glioma.

## Figures and Tables

**Table 1:** Relationship between Girdin expression and clinic-pathological factors of 560 Gliomas.

Variable	*n*	Girdin (*n* (%))		*X* ^2^	*P* value
		+	−		
Sex				2.678	0.102
Male	306	104	202		
Female	254	70	184		
Age (years)				1.470	0.225
≤50	320	106	214		
>50	240	68	172		
KPS				9.469	0.002
≤80	184	73	111		
>80	376	101	275		
Extent of resection				30.097	0.001
Subtotal	128	65	63		
Total	432	109	323		
Ki67				42.426	0.001
+	262	117	145		
−	298	57	241		
WHO garde				4.425	0.035
I-II	203	52	151		
III-IV	357	122	235		

**Table 2:** Multivariate analysis of the factors related to Girdin expression.

Characteristic	Exp(*B*)	95% CI for Exp(*B*)	*P* value
Sex	0.952	0.564–1.607	0.854
Age	1.538	0.828–2.855	0.173
KPS	1.120	0.669–1.876	0.666
Extent of resection	1.748	1.134–2.695	0.011
Ki67	0.263	0.111–0.622	0.002
WHO grade	2.563	1.585–3.161	0.001
Constant	0.022		

CI: confidence interval.

**Table 3:** Cox model regression analysis of prognostic factors for the gliomas.

Varies	OR	95% CI for OR	*P* value
Sex	1.182	0.996–1.403	0.056
Age	0.938	0.751–1.170	0.569
KPS	0.726	0.580–0.909	0.050
Extent of resection	2.159	1.521–3.065	0.001
Ki67	1.805	0.681–0.951	0.011
WHO grade	1.894	1.496–2.816	0.001
GIRDIN	2.295	1.870–2.816	0.001
